# Integrated Care in Prostate Cancer (ICARE-P): Nonrandomized Controlled Feasibility Study of Online Holistic Needs Assessment, Linking the Patient and the Health Care Team

**DOI:** 10.2196/resprot.7667

**Published:** 2017-07-28

**Authors:** Veronica Nanton, Rebecca Appleton, Jeremy Dale, Julia Roscoe, Thomas Hamborg, Sam H Ahmedzai, Theodoros N Arvanitis, Douglas Badger, Nicholas James, Richard Mendelsohn, Omar Khan, Deepak Parashar, Prashant Patel

**Affiliations:** ^1^ Division of Health Sciences Warwick Medical School University of Warwick Coventry United Kingdom; ^2^ Medical School University of Sheffield Sheffield United Kingdom; ^3^ Institute of Digital Healthcare WMG University of Warwick Coventry United Kingdom; ^4^ South Warwickshire Prostate Support Association Stratford United Kingdom; ^5^ The Cancer Centre University Hospital Birmingham NHS Foundation Trust Birmingham United Kingdom; ^6^ Birmingham South Central CCG Birmingham United Kingdom; ^7^ Department of Urology University Hospital Birmingham NHS Foundation Trust Birmingham United Kingdom

**Keywords:** Internet, prostate cancer, holistic needs assessment, integrated care

## Abstract

**Background:**

The potential of technology to aid integration of care delivery systems is being explored in a range of contexts across a variety of conditions in the United Kingdom. Prostate cancer is the most common cancer in UK men. With a 10-year survival rate of 84%, there is a need to explore innovative methods of care that are integrated between primary health care providers and specialist teams in order to address long-term consequences of the disease and its treatment as well as to provide continued monitoring for recurrence.

**Objective:**

Our aim was to test the feasibility of a randomized controlled trial to compare a model of prostate cancer continuing and follow-up care integration, underpinned by digital technology, with usual care in terms of clinical and cost-effectiveness, patient-reported outcomes, and experience.

**Methods:**

A first phase of the study has included development of an online adaptive prostate specific Holistic Needs Assessment system (HNA), training for primary care-based nurses, training of an IT peer supporter, and interviews with health care professionals and men with prostate cancer to explore views of their care, experience of technology, and views of the proposed intervention. In Phase 2, men in the intervention arm will complete the HNA at home to help identify and articulate concerns and share them with their health care professionals, in both primary and specialist care. Participants in the control arm will receive usual care. Outcomes including quality of life and well-being, prostate-specific concerns, and patient enablement will be measured 3 times over a 9-month period.

**Results:**

Findings from phase 1 indicated strong support for the intervention among men, including those who had had little experience of digital technology. Men expressed a range of views on ways that the online system might be used within a clinical pathway. Health care professionals gave valuable feedback on how the output of the assessment might be presented to encourage engagement and uptake by clinical teams. Recruitment to the second phase of the study, the feasibility trial, commenced March 2017.

**Conclusions:**

To our knowledge, this study is the first in the United Kingdom to trial an online holistic needs assessment for men with prostate cancer, with data shared between patients and primary and secondary care providers. This study addresses recommendations in recent policy documents promoting the importance of data sharing and enhanced communication between care providers as a basis for care integration. We anticipate that this model of care will ultimately provide important benefits for both patients and the National Health Service.

**Trial Registration:**

International Standard Randomized Controlled Trial Number (ISRCTN): 31380482; http://www.isrctn.com/ISRCTN31380482 (Archived by WebCite at http://www.webcitation.org/6s8I42u5N)

## Introduction

Increasing numbers of cancer patients and cancer survivors represent a growing demand on overstretched specialist secondary care services in the United Kingdom. Evidence indicates that these services often fail to meet the various needs of survivors [1-4]. The potential role for primary care in addressing these needs and reducing the demand on specialist services is now widely recognized [5]. In response, there has been a rapid increase in recent years in interventions aiming to introduce and evaluate integrated and shared approaches to care, in which primary care services take on an extended role in the care of patients during or following treatment [6,7]. Macmillan Cancer Support has promoted greater primary care involvement with patients with cancer and has developed and rolled out a training program in cancer follow-up for practice nurses [8].

Prostate cancer is the most common cancer in men, with over 47,000 men diagnosed each year in the United Kingdom [9]. With current 10-year survival rates of 84% [10], there are over 330,000 men in the United Kingdom living with and after prostate cancer. Men with prostate cancer follow a range of treatment pathways depending on factors such as the stage their cancer was diagnosed, comorbidities, and patient choice. They often live with the direct consequences of their illness or treatment, such as urinary, sexual and bowel problems, as well as fatigue. These symptoms can have a negative impact on men’s quality of life [11-14]. Men on hormone treatment also face an increased risk of osteoporosis and cardiovascular events [15-18]. In addition, men are often affected by indirect consequences of their cancer such as social, financial, and psychological difficulties [4,19,20]. In particular, men may suffer from anxiety and depression, both during initial treatment and thereafter [21,22]. These may be especially pronounced if they have a lack of positive support, detrimental interactions, and a high perceived threat of cancer [23]. This may contribute to men with prostate cancer reporting a worse experience of care and follow-up than those with other cancers [24,25]. There is also evidence that problems may remain unrecognized and unaddressed and may persist for many years [26-28].

While high survival rates in prostate cancer are extremely encouraging, there remains a need for greater attention to quality of life. As well as treatment of the disease and its physical symptoms, methods to identify men’s emotional and psychological concerns, or “holistic needs,” are required. Care pathways that are designed to address these from an early stage are needed.

### A Role for Primary Care

While protocols vary widely throughout the United Kingdom, the National Institute for Health and Care Excellence (NICE) has recommended that after 2 years of secondary care‒based follow-up, care should be transferred to the general practitioner (GP) as long as there are no concerning symptoms or treatment complications [29]. Many specialist services currently transfer care of men with “stable” disease either after surgery or on hormone treatment to primary care providers under locally enhanced or incentivized service arrangements with provision for men to return to specialist care if necessary. Primary care clinicians appear willing to undertake an early role in follow-up [30-32]. Interventions that increase communication between primary and secondary specialist services have been shown to facilitate the transfer of care process [33].

Practice nurses and other primary care team members are well placed to understand the wide ranging needs of men with prostate cancer with their knowledge of each patient’s comorbidities, family, and social circumstances. They are also better placed to provide opportunistic support, as GP consultation rates for men with prostate cancer are around three times higher than those of their peers [34]. Much of their expertise and skills in the care of patients with chronic conditions in relation to supporting self-management may be transferable to prostate cancer care. Primary care staff may also have detailed knowledge of local resources that may be of value to the patient, and GPs may make direct referrals to community support services. Evaluation within one Clinical Commissioning Group, where follow-up for stable patients has been transferred to primary care, has demonstrated benefits in terms of patient experience and costs and barriers to the pathway redesign including issues around primary and secondary care communication [35].

### Holistic Needs Assessment

In recognition of the changing needs of cancer patients throughout their cancer journey, “holistic needs assessment” as a method of identifying, assessing, and planning appropriate care has been widely promoted for use in the specialist secondary care setting. The National Cancer Survivorship Initiative’s “Living with and beyond cancer programme” recommended that all cancer patients should have access to a Holistic Needs Assessment (HNA) and Care Plan [36]. The program promotes the use of the HNA for encouraging patients to self-manage during and after their treatment. A recent National Prostate Cancer Audit [37] has also highlighted the importance of identifying the needs of men with prostate cancer and linking men to appropriate services. While there is general agreement regarding the potential of the HNA, there is little evidence of effectiveness [38,39]. Adoption of the HNA with respect to men with prostate cancer has also been shown to be uneven, with staff identifying barriers including the time needed for completion in clinic (Prostate Cancer UK unpublished report). To address the limitations of a paper-based system [40], Macmillan Cancer Support has developed an electronic generic HNA. Evaluation has demonstrated acceptability of the electronic format to both patients and staff but pointed out difficulties of implementation within the secondary care setting [41]. To date, there is little indication of uptake of such assessments tools within general practice.

### Information Technology

The use of information technology (IT) in cancer patient follow-up and primary care has been explored in the United States [42], Norway [43,44], and Australia [45] but has as yet received limited attention within the United Kingdom. However, technology now well embedded within general practice management is increasingly incorporated into patient care in a variety of forms and across a range of conditions (eg [6,46-50]). The GP Forward View policy document describing the National Health Service (NHS) plans for developing general practice in the next 5 years emphasizes its increasing importance and includes a key policy aim to support the design and adoption of technology that enables patient self-care and self-management. The document also highlights the central role of technology in improving primary and secondary care communication and the provision of integrated care [51]. Secondary care adoption of IT, however, has been comparatively slow and the need for secondary care digitization, including patient-facing systems, has recently been highlighted by the National Advisory Group on Health Information Technology in England [52].

In order to better address the ongoing needs of men with prostate cancer and to try to reduce the pressures on specialist services, we have designed an innovative study that brings together IT, HNA, and primary care involvement throughout the cancer pathway. Our intervention aims to promote an integrated approach to care through enhanced communication between the patient and health care providers. This protocol describes a feasibility study in which an online prostate-specific HNA is shared between patient and their clinical teams. In addition, our study involves training for practice nurses in collaboration with Macmillan Cancer Support and ongoing support from specialist teams. To our knowledge, this is the first UK-based study in which IT provides the basis for a primary care‒based intervention in prostate cancer and the first UK-based study that seeks to develop an integrated and holistic approach to care for all men with prostate cancer from diagnosis onwards.

## Methods

### Design

This study involves a complex intervention [53] that includes both the development of patient self-efficacy and the implementation of organizational change [54]. Hence, we have adopted a mixed-methods, two-phase design. Phase 1 of the study (March-November 2016) was designed to include technical development, training, recruitment, and qualitative interviews. Phase 2 is a nonrandomized cluster controlled trial of the intervention. Follow-up interviews will be undertaken with patient participants and health professionals to explore their experience of the trial.

### Population, Setting, and Inclusion Criteria

Our study population extends to all men who have ever had a diagnosis of prostate cancer. The selection of this population offers the opportunity for participation to any man who may have problems related to prostate cancer, whether currently receiving treatment, monitoring, or follow-up. This will allow us to identify the wide range of short- and long-term concerns that may occur during or following different treatment regimens.

The study is set in 14 general practices including 10 intervention and 4 control practices within one Clinical Commissioning Group (CCG) in the West Midlands and one specialist secondary care site within an NHS Foundation Trust hospital. Eligibility for general practices is determined by location within the participating CCG and a referral pathway to the specialist center. Practices must also be willing to support a practice nurse taking part in the Macmillan training program and the prostate specific training or to enable a primary care research nurse who has undergone the training to run the study within the practice.

Patient eligibility for each of the study phases requires registration with any of the participating practices and diagnosis of prostate cancer or treatment at any time at the participating specialist center. Men must be able to read written and understand spoken English and be able and willing to give informed consent. GPs’ screening of the list of potentially eligible men ensures capacity to participate.

Men are ineligible for inclusion if they are aged under 18 years, unable to give informed consent, unable to complete outcome measures, living in a care setting, suffer from mental health problems, or are unable to communicate in English.

Practice nurses and GPs from participating practices are eligible to take part in interviews prior to or following the intervention.

### Phase 1 Summary

During Phase 1, the HNA has been finalized and installed on a secure study website at the participating Trust. Participating patients, associated clinicians in secondary and primary care, and members of the study team have access to the site. Ten intervention and 4 control general practices within the participating CCG have been recruited. Five Macmillan trained nurses recruited to the study have undertaken additional prostate cancer training at the specialist secondary care center. One volunteer peer supporter has been recruited and trained in order to help men complete the HNA if needed. Qualitative semistructured interviews have been undertaken with 8 primary care‒based health care professionals and 10 patient participants recruited through their general practices. The purpose of the interviews was to determine variation in delivery and experience of usual care and to identify barriers and facilitators to implementation of the intervention and to assess the views of health care professionals and patients on the online HNA.

Findings from Phase 1 demonstrate an enthusiasm for the project among patients. Men who have had little experience with IT have expressed a willingness to take part, in some cases despite a lack of access to the Internet. We have responded to this potential barrier by equipping the ITmate peer supporter with an Internet-enabled tablet computer. Health professionals have also been supportive of the study and acknowledged the potential of the intervention. GPs in particular expressed the importance of a brief output from the HNA, hence the summary document generated by the assessment has been designed to be concise and easily interpreted.

### Aims and Objectives

The aim of the trial is to test the feasibility of undertaking a future cluster randomized controlled trial (RCT) comparing a model of integrated prostate cancer continuing care and follow-up care with usual care in terms of clinical and cost-effectiveness, patient-reported outcomes, and experience.

Trial objectives are to (1) assess the fidelity of intervention delivery, and the acceptability and utility of the intervention by patients and primary and secondary care clinicians, (2) test patient recruitment, data collection, and retention in both intervention and control practices, (3) analyze how the HNA is used by men over a 9-month period, with self-assessment completed at baseline, 3 months, and 6 months, (4) test the willingness of clinicians and patients to provide qualitative and quantitative data including process data and measurement of outcomes, (5) test the feasibility of collecting use of resource data and process data, including numbers of primary and specialist contacts and consultations, (6) identify the most suitable primary outcome measure and refine secondary outcome measures for a future cluster RCT, and (7) estimate parameters for a sample size calculation for cluster RCT.

### The Holistic Needs Assessment Instrument

The HNA is an online self-assessment tool designed to capture the needs of men who have had a diagnosis of prostate cancer. It is composed of 11 different sections, including Physical Health, Emotional and Psychological Issues, Independence and Activity, and Access to Services. These can be used flexibly so that men can complete all of the sections or only the ones they feel are relevant to them, in any order, and in their own time. As men work through the assessment, they are shown links to advice pages or videos on websites such as Prostate Cancer UK, with the aim of encouraging and enabling self-management. This system shows a “red flag” if a patient reports having any physical or psychological symptoms of serious concern, for example, “blood in urine” or “thoughts of ending it all” with a prompt to visit their GP as soon as possible. The system will also alert the clinician to these red flags when they access the output from their patient’s HNA. At the end of each section, men have the facility to disclose additional concerns, and this text is inserted into the summary generated on completion of the assessment. This summary auto-populates the first pages of a Care Plan to be completed with a health professional.

**Figure 1:**
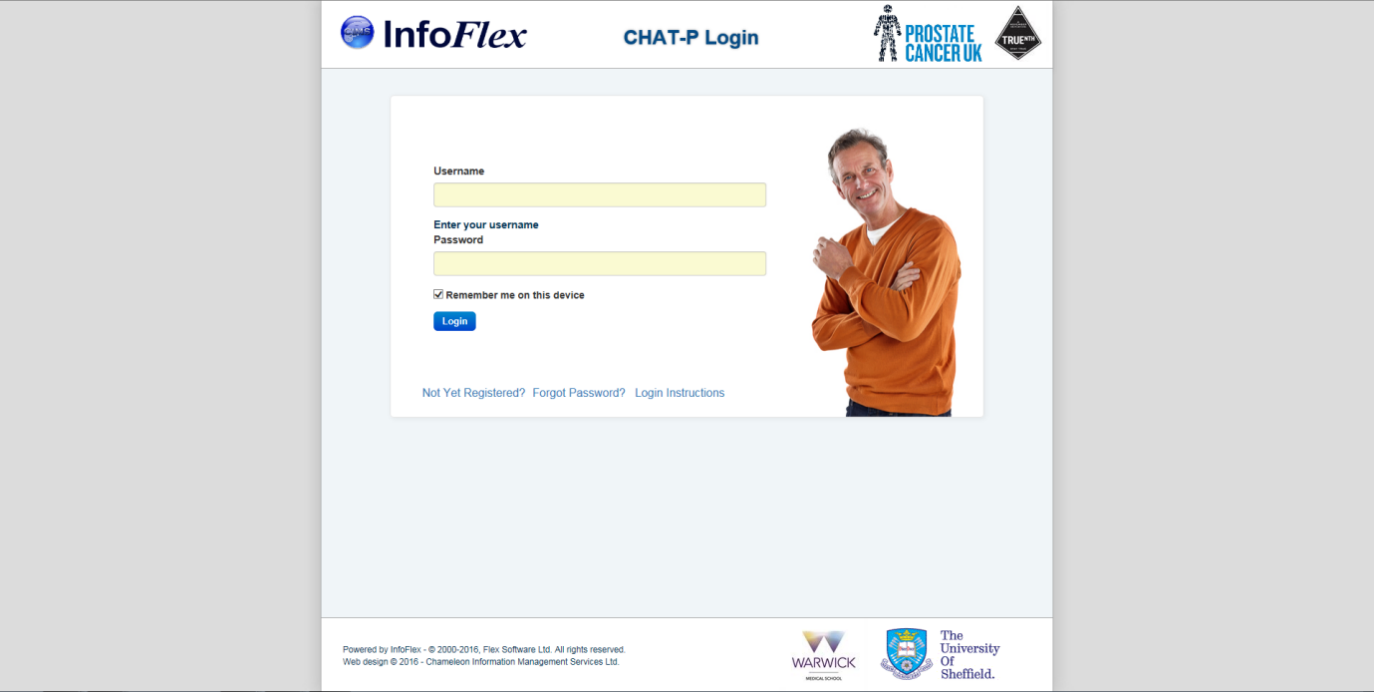
The HNA login screen.

**Figure 2:**
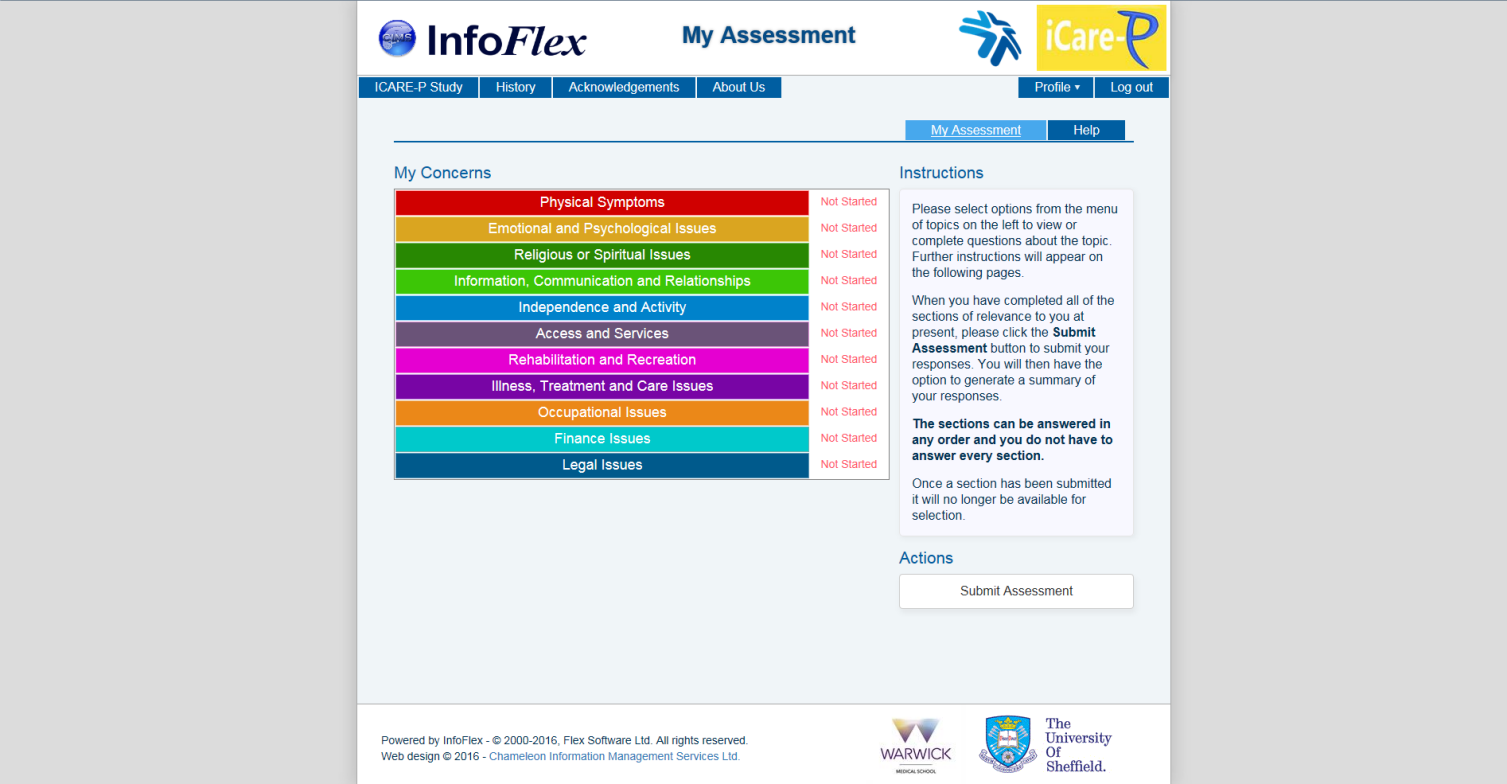
Assessment page showing the different sections of the HNA.

The system has been designed to be attractive and easy to navigate. Figures 1 and 2 illustrate two of the initial screens. The online HNA has been finalized following an iterative process of user and patient testing (Prostate Cancer UK unpublished report). It has been installed on a study website hosted on a Trust server and penetration tested to ensure security.

The project platform incorporates three elements: (1) a Web app for patients to complete the holistic needs assessments, (2) a Web app for nurses to review those assessments and record care plans, and (3) a windows app design to allow administration of the project such as adding patient or nurse users and monitoring completion of study stages by the research team.

### Intervention

Participants in the intervention group will be invited to complete the HNA three times in a 9-month period. Following submission of the assessment, men will be invited to make an appointment with the nurse to discuss any concerns identified and to complete and personalize the care plan. The care plan will summarize any topics discussed, outcomes or referrals, actions taken regarding any “red flag” symptoms, and actions to be undertaken by the man himself. The document will be generated and a copy given to participants and added to their patient notes. Where specific clinical concerns are identified, sharing of the online data with the secondary care team will enable rapid specialist advice or referral. If the participants attend the surgery between the three study appointments, practice nurses or GPs will be encouraged to undertake opportunistic reinforcement of the care plan, for example, checking whether the man has undertaken any actions he has agreed to in the plan or whether referrals made have led to appointments.

If the participant is a patient undergoing ongoing treatment at the specialist center, study participation will be flagged in his notes. Relevant secondary care clinicians will be able to access the participants assessment summary and care plan prior to or during an appointment. If any concerns other than clinical issues related to the prostate cancer arise in the consultation (eg, occupational or family concerns, worries over comorbidities), the secondary care clinician may, with the participant’s permission, contact the primary care‒based nurse.

### Usual Care

Usual care comprises a community-based, stable prostate clinical care pathway. Men with advanced disease on hormone treatment are also largely seen in primary care for their injections and will be seen only in secondary care if their prostate specific antigen (PSA) rises above a threshold or other complications occur. Care of men by specialist teams continues up until 3 years postcurative treatment and is reintroduced if a clinical need arises (symptoms that are suggestive of prostate cancer progression, eg, significant rise in PSA, suspected metastases, pain or treatment complication, deteriorating renal function). Men on active surveillance are excluded from this pathway. Men who do not meet the criteria continue to be seen by secondary care teams.

### Recruitment

The GP and practice nurse will search for eligible patients, who will then be sent a participant information sheet and a covering letter of invitation to participate signed by the GP. If patients are considering taking part in the study or have decided to do so, they will return an enclosed reply slip to the study team. If there has been no response after 2 weeks from the initial contact, the practice nurse will telephone to check whether the patient is interested. Patients can also be invited to take part on an opportunistic basis, such as during a routine appointment. Written consent will be taken by a member of the study team.

### Data Collection

Participants in the intervention group will complete the HNA at baseline, as well as 3 months and 6 months later. We will use six validated questionnaires to measure unmet need, quality of life, and general health and well-being of participants: the Cancer Survivors Unmet Needs instrument [55], the Patient Activation Measure [56], the Expanded Prostate Cancer Index Composite [57], the EuroQol five dimensions questionnaire (EQ5D) [58], the European Organisation for Research and Treatment of Cancer Quality of Life Questionnaire (EORTC-QLQ) [59], and the Warwick and Edinburgh Mental Well-being Scale [60], as participants in the intervention group progress through the study. At baseline and 9 months later, participants in both the intervention and control groups will complete all the outcome measures. Participants in the intervention group will also complete a smaller subset of outcome measures before consultations at 3 and 6 months. Men can either complete these online or fill out paper copies for postal return.

A small number of patients from the intervention group (n<10) will be followed up at the end of the study for an interview about their experiences of the intervention. We will also invite participants to complete an HNA technology acceptance and usability questionnaire developed by members of the study team.

### Outcomes

Patient and feasibility outcomes will be assessed. In order to determine feasibility of carrying out a larger scale RCT to compare the intervention with usual care, we will evaluate this study in terms of >25% eligible men consenting to participate in the study and >70% of participants in the intervention group completing the HNA and the patient outcome measures at each time point.

**Figure 3:**
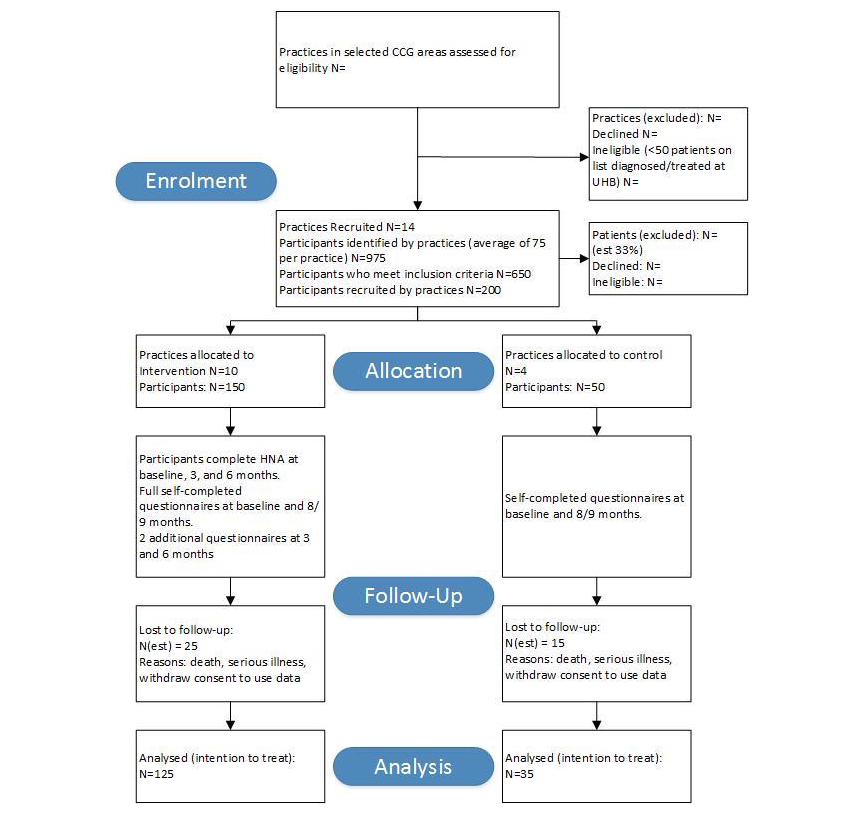
Flowchart for ICARE-P Adapted from CONSORT guidelines for randomized controlled trials.

Figure 3 illustrates the study pathway.

### Analysis

#### Quantitative

The analyses will be exploratory and mainly descriptive. Point estimates and corresponding 90% confidence intervals will be calculated for all outcome measures in both arms, and their distributions will be assessed to identify appropriate statistical analysis methods for a future RCT. Hierarchical mixed models accounting for within GP practice correlation will be fitted for potential primary outcomes to explore the effect of the intervention relative to usual care at a single time point as well as over time (repeated measures model). Propensity score matching and regression adjustment techniques will be used as sensitivity analyses and to minimize potential bias in outcomes estimates due to the nonrandomized nature of the study.

Data will be downloaded from the HNA database to assess completion rates at each time period, sequential completion over a year, and analysis of the needs identified.

#### Qualitative

Thematic analysis of the interviews will also take place with the audiorecorded data transcribed and entered into a software analysis package (NVivo 10). Members of the research team will each identify broad themes from close reading of the interview transcripts. Further themes and subthemes will be developed through an iterative process of coding, categorization, and discussion between team members. Topics to be explored include men’s experiences of the new model of care, advantages and disadvantages of the intervention, and any impact on heath and confidence in self-management.

#### Health Economic Analysis

Completion rates for collecting resource utilization data, contacts with health care professionals and referrals to hospitals and other community health and social care providers will be evaluated. Data collected at each time point will be analyzed to provide preliminary indications of the costs associated with the intervention over the 9-month period.

#### Intervention Fidelity

Researchers within the project team are able to access the ICARE-P admin site. This will allow the tracking of completion of HNAs and Care Plans and to identify how the software has been used by participants and health professionals. Content of the HNAs and Care Plans are also accessible to these members of the research team. Analysis of Care Plans and referral data retrieved from practices will allow assessment of intervention fidelity.

### Ethics

Phase 1 of the study was approved by NHS Research Ethics Committee proportionate review process (ref: 15/EM/0534). Phase 2 was considered and approved under full review (ref: 16/YH/0278).

## Discussion

### Principal Considerations

The online HNA that forms the cornerstone of our intervention puts the patient at the center of care [61], enabling him to identify and express concerns to both primary care and specialist teams, as well as encouraging and facilitating self-management. The adaptive nature of the HNA ensures its suitability for men from early diagnosis onwards regardless of treatment modality or their stage in the care pathway. The online system offers an opportunity for integrated care allowing all linked clinicians access to the HNA output in which prostate specific clinical issues, broader concerns, or issues related to comorbidities are summarized.

Integration between primary and specialist secondary care services and the role of technology in supporting this aim represent key themes of the five-year forward policy document [51]. Integrated services offer advantages to patients in terms of continuity and coordination together with a potential for streamlining of provision. Interoperability of systems that allow easy data sharing are critical to integration, and the implementation of such systems has been identified as priority in a recent report by the National Advisory Group on Health Information Technology in England [52]. Primary care clinicians interviewed in Phase 1 of our study endorsed the need for improvements in communication with specialist colleagues, expressing frustration at the speed of current systems. The report has also highlighted the relatively slow adoption of digital technology in secondary care compared with high levels of primary care digitization and has emphasized the need for secondary care implementation. Barriers identified include extreme caution in relation to data security and a lack of attention to the needs of the end user when systems have been developed and put in place. Our HNA and Care Plan have been designed for ease of use by patients and clinicians, and they aim to be comprehensive yet relevant. We have provided training for participating clinicians in the use of the system to encourage engagement with the study. To overcome any difficulties patients may experience, we have the trained ITmate available to assist them. We have met the need to conform to rigorous data security standards and have worked collaboratively with our participating NHS Trust, our institution, and our IT partners to ensure all requirements are met.

A further recommendation of the report is a stepwise approach to digitization. We recognize the importance of such an approach both from the technological and the behavioral standpoints. This study introduces an innovative approach to the care of men with prostate cancer; however, more can be achieved.

If feasibility aims are met in terms of recruitment (>25% of eligible men and >70% retention) and usability and acceptability testing with patients and clinical teams indicate support for the intervention, further technical development of the HNA is proposed prior to a full effectiveness trial. This development will involve full integration of the HNA within the Trusts’ own clinical data management system and the development of an enhanced communication pathway between specialist and primary care teams enabling automated alerts and notifications.

### Limitations

As a feasibility study, the sample size involved is limited. A nonrandomized controlled trial may result in some biases (in particular confounding and allocation bias). We aim to reduce these biases through the use of regression adjustment and propensity score matching.

### Conclusion

This study is the first of its kind to trial an online HNA for men with prostate cancer with data shared between patients and primary and secondary care providers. We anticipate that this system will ultimately provide important benefits for patients in terms of addressing unmet needs, identifying concerning symptoms, and enabling self-management, and benefits to the NHS in terms of effective use of resources and appropriate use of skills.

## Abbreviations

GP: general practitioner
HNA: holistic needs assessment
ICARE-P: integrated system to improve patient outcomes and experience
IT: information technology
NHS: National Health Service
NICE: National Institute for Health and Care Excellence
RCT: randomized controlled trial

## References

[1] Richards M, Corner J, Maher J (2011). The National Cancer Survivorship Initiative: new and emerging evidence on the ongoing needs of cancer survivors. Br J Cancer.

[2] Davies NJ, Batehup L (2011). Towards a personalised approach to aftercare: a review of cancer follow-up in the UK. J Cancer Surviv.

[3] Carey M, Lambert S, Smits R, Paul C, Sanson-Fisher R, Clinton-McHarg T (2012). The unfulfilled promise: a systematic review of interventions to reduce the unmet supportive care needs of cancer patients. Support Care Cancer.

[4] Ream E, Quennell A, Fincham L, Faithfull S, Khoo V, Wilson-Barnett J, Richardson A (2008). Supportive care needs of men living with prostate cancer in England: a survey. Br J Cancer.

[5] Buckland D (2016). Role of primary care in the management of cancer patients. Prescriber.

[6] Tomasone JR, Brouwers MC, Vukmirovic M, Grunfeld E, O'Brien MA, Urquhart R, Walker M, Webster F, Fitch M (2016). Interventions to improve care coordination between primary healthcare and oncology care providers: a systematic review. ESMO Open.

[7] Kendall M, Mason B, Momen N, Barclay S, Munday D, Lovick R, Macpherson S, Paterson E, Baughan P, Cormie P, Kiehlmann P, Free A, Murray SA (2013). Proactive cancer care in primary care: a mixed-methods study. Fam Pract.

[8] Macmillan (2013). Macmillan UK.

[9] Prostate Cancer UK.

[10] Cancer Research UK.

[11] Watson E, Shinkins B, Frith E, Neal D, Hamdy F, Walter F, Weller D, Wilkinson C, Faithfull S, Wolstenholme J, Sooriakumaran P, Kastner C, Campbell C, Neal R, Butcher H, Matthews M, Perera R, Rose P (2016). Symptoms, unmet needs, psychological well-being and health status in survivors of prostate cancer: implications for redesigning follow-up. BJU Int.

[12] Pardo Y, Guedea F, Aguiló F, Fernández P, Macías V, Mariño A, Hervás A, Herruzo I, Ortiz MJ, Ponce de León J, Craven-Bratle J, Suárez JF, Boladeras A, Pont A, Ayala A, Sancho G, Martínez E, Alonso J, Ferrer M (2010). Quality-of-life impact of primary treatments for localized prostate cancer in patients without hormonal treatment. J Clin Oncol.

[13] Gavin AT, Drummond FJ, Donnelly C, O'Leary E, Sharp L, Kinnear HR (2015). Patient-reported 'ever had' and 'current' long-term physical symptoms after prostate cancer treatments. BJU Int.

[14] Lehto U, Helander S, Taari K, Aromaa A (2015). Patient experiences at diagnosis and psychological well-being in prostate cancer: A Finnish national survey. Eur J Oncol Nurs.

[15] Potosky AL, Davis WW, Hoffman RM, Stanford JL, Stephenson RA, Penson DF, Harlan LC (2004). Five-year outcomes after prostatectomy or radiotherapy for prostate cancer: the prostate cancer outcomes study. J Natl Cancer Inst.

[16] Resnick MJ, Koyama T, Fan K, Albertsen PC, Goodman M, Hamilton AS, Hoffman RM, Potosky AL, Stanford JL, Stroup AM, Van Horn RL, Penson DF (2013). Long-term functional outcomes after treatment for localized prostate cancer. N Engl J Med.

[17] Daniell HW, Dunn SR, Ferguson DW, Lomas G, Niazi Z, Stratte PT (2000). Progressive osteoporosis during androgen deprivation therapy for prostate cancer. J Urol.

[18] Nguyen PL, Je Y, Schutz FAB, Hoffman KE, Hu JC, Parekh A, Beckman JA, Choueiri TK (2011). Association of androgen deprivation therapy with cardiovascular death in patients with prostate cancer: a meta-analysis of randomized trials. JAMA.

[19] Armes J, Crowe M, Colbourne L, Morgan H, Murrells T, Oakley C, Palmer N, Ream E, Young A, Richardson A (2009). Patients' supportive care needs beyond the end of cancer treatment: a prospective, longitudinal survey. J Clin Oncol.

[20] Weber BA, Sherwill-Navarro P (2005). Psychosocial consequences of prostate cancer: 30 years of research. Geriatr Nurs.

[21] Sharpley CF, Christie DRH (2007). Patient information preferences among breast and prostate cancer patients. Australas Radiol.

[22] Korfage IJ, Essink-Bot M, Janssens ACJW, Schröder FH, de Koning HJ (2006). Anxiety and depression after prostate cancer diagnosis and treatment: 5-year follow-up. Br J Cancer.

[23] Mehnert A, Lehmann C, Graefen M, Huland H, Koch U (2010). Depression, anxiety, post-traumatic stress disorder and health-related quality of life and its association with social support in ambulatory prostate cancer patients. Eur J Cancer Care (Engl).

[24] Sinfield P, Baker R, Camosso‐Stefinovic J, Colman A, Tarrant C, Mellon J, Steward W, Kockelbergh R, Agarwal S (2009). Men’s and carers’ experiences of care for prostate cancer: a narrative literature review. Health Expect.

[25] (2012). Department of Health.

[26] Nanton V, Docherty A, Meystre C, Dale J (2009). Finding a pathway: information and uncertainty along the prostate cancer patient journey. Br J Health Psychol.

[27] O'Brien R, Rose P, Campbell C, Weller D, Neal RD, Wilkinson C, McIntosh H, Watson E, Prostate Cancer Follow-up Group (2011). “I wish I'd told them”: a qualitative study examining the unmet psychosexual needs of prostate cancer patients during follow-up after treatment. Patient Educ Couns.

[28] Morrison V, Henderson BJ, Zinovieff F, Davies G, Cartmell R, Hall A, Gollins S (2012). Common, important, and unmet needs of cancer outpatients. Eur J Oncol Nurs.

[29] (2014). NICE.

[30] Watson EK, O'Brien R, Campbell C, Weller D, Neal RD, Wilkinson C, Rose PW, Prostate Cancer Follow-Up Study Group (2011). Views of health professionals on the role of primary care in the follow-up of men with prostate cancer. Fam Pract.

[31] Del Giudice ME, Grunfeld E, Harvey BJ, Piliotis E, Verma S (2009). Primary care physicians' views of routine follow-up care of cancer survivors. J Clin Oncol.

[32] Jiwa M, McManus A, Dadich A (2013). The impact of knowledge, attitudes and beliefs on the engagement of primary and community-based healthcare professionals in cancer care: a literature review. Curr Med Res Opin.

[33] Lewis RA, Neal RD, Williams NH, France B, Hendry M, Russell D, Hughes DA, Russell I, Stuart NSA, Weller D, Wilkinson C (2009). Follow-up of cancer in primary care versus secondary care: systematic review. Br J Gen Pract.

[34] Heins M, Schellevis F, Rijken M, van der Hoek L, Korevaar J (2012). Determinants of increased primary health care use in cancer survivors. J Clin Oncol.

[35] NHS Transforming Cancer Services for London Team (2016). NHS.

[36] (2013). Department of Health.

[37] (2016). National Prostate Cancer Audit.

[38] Richardson A, Medina J, Brown V, Sitzia J (2007). Patients' needs assessment in cancer care: a review of assessment tools. Support Care Cancer.

[39] Carlson LE, Waller A, Mitchell AJ (2012). Screening for distress and unmet needs in patients with cancer: review and recommendations. J Clin Oncol.

[40] Richardson AS, Brown V, Medina JA (2005). Patients' Needs Assessment Tools in Cancer Care: Principles and Practice.

[41] (2013). IPSOS Mori.

[42] Vickers AJ, Salz T, Basch E, Cooperberg MR, Carroll PR, Tighe F, Eastham J, Rosen RC (2010). Electronic patient self-assessment and management (SAM): a novel framework for cancer survivorship. BMC Med Inform Decis Mak.

[43] Ruland CM, Andersen T, Jeneson A, Moore S, Grimsbø GH, Børøsund E, Ellison MC (2013). Effects of an internet support system to assist cancer patients in reducing symptom distress: a randomized controlled trial. Cancer Nurs.

[44] Wibe T, Hellesø R, Varsi C, Ruland C, Ekstedt M (2012). How does an online patient-nurse communication service meet the information needs of men with recently diagnosed testicular cancer?. ISRN Nurs.

[45] Jiwa M, McManus A, Dadich A, White J, Rieck A, Razmi S (2013). Harnessing information technology to innovate in primary care. Qual Prim Care.

[46] Young AJ (2016). New technologies and general practice. Br J Gen Pract.

[47] Bodenheimer T, Grumbach K (2003). Electronic technology: a spark to revitalize primary care?. JAMA.

[48] Meade B, Buckley D, Boland M (2009). What factors affect the use of electronic patient records by Irish GPs?. Int J Med Inform.

[49] McMillan B, Abdelgalil R, Madhuvrata P, Easton K, Mitchell C (2016). Reducing the risk of type 2 diabetes mellitus in primary care after gestational diabetes: a role for mobile technology to improve current care. Br J Gen Pract.

[50] Smith JJ, Mallard-Smith RJ, Beattie V, Beattie DK (2003). Use of information technology in general practice. J R Soc Med.

[51] (2016). NHS England.

[52] Wachter R (2016). Department of Health.

[53] Anderson R (2008). New MRC guidance on evaluating complex interventions. BMJ.

[54] Plamping D (1998). The NHS's 50 anniversary. Change and resistance to change in the NHS. BMJ.

[55] Hodgkinson K, Butow P, Hunt GE, Pendlebury S, Hobbs KM, Lo SK, Wain G (2007). The development and evaluation of a measure to assess cancer survivors' unmet supportive care needs: the CaSUN (Cancer Survivors' Unmet Needs measure). Psychooncology.

[56] Hibbard JH, Stockard J, Mahoney ER, Tusler M (2004). Development of the Patient Activation Measure (PAM): conceptualizing and measuring activation in patients and consumers. Health Serv Res.

[57] Chang P, Szymanski KM, Dunn RL, Chipman JJ, Litwin MS, Nguyen PL, Sweeney CJ, Cook R, Wagner AA, DeWolf WC, Bubley GJ, Funches R, Aronovitz JA, Wei JT, Sanda MG (2011). Expanded prostate cancer index composite for clinical practice: development and validation of a practical health related quality of life instrument for use in the routine clinical care of patients with prostate cancer. J Urol.

[58] Herdman M, Gudex C, Lloyd A, Janssen M, Kind P, Parkin D, Bonsel G, Badia X (2011). Development and preliminary testing of the new five-level version of EQ-5D (EQ-5D-5L). Qual Life Res.

[59] Groenvold M, Klee MC, Sprangers MA, Aaronson NK (1997). Validation of the EORTC QLQ-C30 quality of life questionnaire through combined qualitative and quantitative assessment of patient-observer agreement. J Clin Epidemiol.

[60] Tennant R, Hiller L, Fishwick R, Platt S, Joseph S, Weich S, Parkinson J, Secker J, Stewart-Brown S (2007). The Warwick-Edinburgh Mental Well-being Scale (WEMWBS): development and UK validation. Health Qual Life Outcomes.

[61] Dunn N (2003). Practical issues around putting the patient at the centre of care. J R Soc Med.

